# LFA-1 Controls Th1 and Th17 Motility Behavior in the Inflamed Central Nervous System

**DOI:** 10.3389/fimmu.2019.02436

**Published:** 2019-10-18

**Authors:** Silvia Dusi, Stefano Angiari, Enrica Caterina Pietronigro, Nicola Lopez, Gabriele Angelini, Elena Zenaro, Vittorina Della Bianca, Gabriele Tosadori, Francesca Paris, Antonella Amoruso, Tommaso Carlucci, Gabriela Constantin, Barbara Rossi

**Affiliations:** ^1^Department of Medicine, Section of General Pathology, University of Verona, Verona, Italy; ^2^The Center for Biomedical Computing (CBMC), University of Verona, Verona, Italy

**Keywords:** lymphocyte function-associated antigen 1, Th1 and Th17 cells, intra-tissue motility, two-photon laser microscopy, experimental autoimmune encephalomyelitis

## Abstract

Leukocyte trafficking is a key event during autoimmune and inflammatory responses. The subarachnoid space (SAS) and cerebrospinal fluid are major routes for the migration of encephalitogenic T cells into the central nervous system (CNS) during experimental autoimmune encephalomyelitis (EAE), the animal model of multiple sclerosis, and are sites of T cell activation before the invasion of CNS parenchyma. In particular, autoreactive Th1 and Th17 cell trafficking and reactivation in the CNS are required for the pathogenesis of EAE. However, the molecular mechanisms controlling T cell dynamics during EAE are unclear. We used two-photon laser microscopy to show that autoreactive Th1 and Th17 cells display distinct motility behavior within the SAS in the spinal cords of mice immunized with the myelin oligodendrocyte glycoprotein peptide MOG_35−55_. Th1 cells showed a strong directional bias at the disease peak, moving in a straight line and covering long distances, whereas Th17 cells exhibited more constrained motility. The dynamics of both Th1 and Th17 cells were strongly affected by blocking the integrin LFA-1, which interfered with the deformability and biomechanics of Th1 but not Th17 cells. The intrathecal injection of a blocking anti-LFA-1 antibody at the onset of disease significantly inhibited EAE progression and also strongly reduced neuro-inflammation in the immunized mice. Our results show that LFA-1 plays a pivotal role in T cell motility during EAE and suggest that interfering with the molecular mechanisms controlling T cell motility can help to reduce the pathogenic potential of autoreactive lymphocytes.

## Introduction

Multiple sclerosis (MS), and its animal model experimental autoimmune encephalomyelitis (EAE), is now generally accepted as an inflammatory dysfunction with evidence supporting an autoimmune basis. In particular, leukocyte trafficking from the peripheral circulation to the central nervous system (CNS) induces tissue damage and activates resident glial cells, disrupting myelin sheaths and destroying the underlying axons (1). The analysis of T cells infiltrating the CNS during EAE revealed the presence of both Th1 and Th17 cells, which are potent mediators of inflammation and autoimmunity (2, 3). In MS patients, both Th1 and Th17 cells seem to be involved in disease onset and progression, but their relative contribution to the pathologies of EAE and MS are not yet well defined (1, 4).

Th1 and Th17 cells may play different roles during autoinflammatory diseases. In particular, the relative proportion of Th1 and Th17 cells migrating into the CNS varies among different mouse strains during EAE. Autoreactive Th1 cells were prevalent in the inflamed CNS at the disease peak when EAE was induced by immunizing C57BL/6 mice with myelin oligodendrocyte glycoprotein (MOG), whereas Th17 cells were prevalent at the same stage in Swiss Jim Lambert (SJL) mice immunized with proteolipid peptide (2, 3). Furthermore, the immunization of mice with distinct MOG epitopes elicited T cell responses with different Th1:Th17 ratios, depending on the avidity of T cells for their cognate antigen (5). These data suggest that the relative contribution of Th1 and Th17 cell responses during CNS inflammation might vary depending on the strain of mice and the immunization strategy. Another important feature is the distinct pathologies induced by Th1 and Th17 cells. Th1-mediated CNS inflammation is characterized by infiltrating macrophages, whereas neutrophils are more prevalent when Th17 cells are used to induce the disease (6). Th1 and Th17 cells have a similar capacity to induce EAE, although they differ in terms of the localization of CNS lesions and the clinical disease symptoms (5). The transfer of autoreactive Th17 cells mainly induced brain inflammation in recipient mice, which developed atypical EAE, whereas the transfer of Th1 cells led to the development of classical EAE with predominant spinal cord inflammation. Studies in MS patients and normal human donors have provided evidence that Th1 and Th17 cells are active, particularly during the active disease phase, but their contribution to the heterogeneous nature of the disease is unclear (7–10).

Two-photon laser microscopy (TPLM) has helped to characterize the dynamic behavior of autoreactive effector T cells in the CNS during EAE. The subarachnoid space (SAS) and cerebrospinal fluid (CSF) are preferential routes of entry for the recruitment of T cells into the CNS during early phases of EAE. Here, local antigen-presenting cells (APCs) display endogenous antigens and activate the infiltrated T cells, guiding them into the CNS parenchyma (11–14). Having reached the CNS, exogenous myelin-specific CD4^+^ T cells injected into recipient EAE animals are reactivated inside the spinal cord parenchyma by resident and infiltrating APCs, sustaining the inflammatory process and promoting demyelination and axonal damage (15–17). As seen in the secondary lymphoid organs, where leukocyte motility facilitates the efficient and rapid screening of professional APCs (18), CD4^+^ T cell motility inside the SAS could favor the reactivation of encephalitogenic T cells, a critical process in the pathogenesis of MS and EAE. However, differences in motility behavior between Th1 and Th17 cells inside the spinal SAS during EAE have not been investigated, and the molecular mechanisms controlling such processes remain unknown.

Integrins are heterodimeric transmembrane adhesion molecules involved in leukocyte trafficking and are expressed on the surface of a wide range of cell types in an inactive state. Integrin–ligand binding is rapidly regulated by conformational changes, as well as transcriptional induction and redistribution from intracellular pools (19). The best-characterized β_2_-integrin involved in leukocyte recruitment is LFA-1 (lymphocyte function-associated antigen 1, also known as integrin α_L_β_2_), which participates in the rolling of circulating leukocytes on the blood vessel wall, but predominantly mediates their firm arrest on the vascular bed of lymphoid organs and at sites of inflammation via interaction with its ligands, such as intercellular adhesion molecule 1 (ICAM-1) (20). TPLM analysis has revealed that LFA-1–ICAM-1 interactions also play a role in T cell firm arrest during antigen recognition in lymph nodes and modulate the amoeboid migration of T cells, facilitating antigen scanning (21). However, the role of LFA-1 in the motility of T cells during inflammation in the spinal SAS of EAE mice is still unknown.

Here we took an established method for the stable imaging of the mouse spinal SAS (22) and adapted it for the lumbar region, the main site of inflammation in C57BL/6 mice during EAE, allowing stable imaging in living mice. We identified different motility behaviors in Th1 and Th17 cells in the spinal SAS of EAE mice and investigated the ability of LFA-1 to control cell dynamics at the disease peak. Local anti-LFA-1 treatment inhibited disease progression in EAE mice, supporting the view that interfering with the local intra-tissue dynamics of effector T cells may offer a valuable approach for the treatment of autoimmune and inflammatory diseases of the CNS.

## Materials and Methods

### Ethics Statement

All animal experiments were approved by the Italian Ministry of Health, Department of Veterinary Public Health, Nutrition and Food Safety, Directorate General of Animal Health and Veterinary Medicine, as required by Italian legislation (D. Lgs 26/2014, application of European Directive 2010/63/EU). Protocol numbers 33588 and 30969 were assigned for the approved animal experiments.

### Mice

C57BL/6J wild-type (WT) mice and C57BL/6-Tg(Tcra2D2,Tcrb2D2)1Kuch/J (2D2 TCR) transgenic mice carrying a T cell receptor specific for the MOG_35−55_ peptide (23) were obtained from The Jackson Laboratory. Animals were housed under standardized conditions with a 12-h photoperiod in climate-controlled facilities, and were provided with food and water *ad libitum*. All animal experiments were supervised by the local Institutional Animal Care Committee (OPBA) of the University of Verona and were conducted according to current European Community rules.

### Production of MOG_35−55_-Specific Th1 and Th17 Cells

Spleens were collected from 2D2 TCR transgenic mice, and a single-cell suspension was prepared. Spleen cells were cultured as previously described (24). Briefly, 20 × 10^6^ cells per well were cultured in a six-well plate for 7 days in the presence of 20 μg/ml MOG_35−55_ peptide (GenScript Corporation). For Th1 polarization, we added 1 ng/ml IL-12 (R&D Systems) and 10 μg/ml anti-IL-4 antibody (clone 11B11, hybridoma provided by E. C. Butcher, Stanford University). For Th17 cell polarization, we added 5 ng/ml TGFβ, 20 ng/ml IL-6 and 20 ng/ml IL-23 (all from Miltenyi Biotech or R&D Systems), as well as 10 μg/ml anti-IL-4 antibody (as above) and 10 μg/ml anti-IFNγ antibody (clone HB170, R&D Systems). Th1 cells were supplemented with IL-2 (20 U/ml) and Th17 cells with IL-7 (10 ng/ml) after 4 days in culture. After a further 72 h, MOG_35−55_-specific Th1 and Th17 cells were isolated using a Ficoll-Paque density gradient (GE Healthcare Life Sciences) and frozen in fetal calf serum (FCS, Lonza) containing 10% dimethylsulfoxide (DMSO, Sigma-Aldrich). Before use, the cells were thawed and re-stimulated for 3 days in the presence of irradiated splenocytes as APCs (APC:T cell ratio = 5:1) with the same peptide/cytokine cocktail as described above. Th1 and Th17 cells were then isolated using a Ficoll-Paque gradient and supplemented for one further day with IL-2 or IL-7, as appropriate.

### TPLM Preparation

Th1 or Th17 cells were labeled for 45 min at 37°C with 40 mM CMAC or carboxyfluorescein-diacetate-succinimidyl-ester (CFSE) (both from Molecular Probes). We then injected 2 × 10^7^ Th1 or Th17 cells into the tail veins of MOG_35−55_-immunized EAE recipient mice at different disease phases. In order to ensure that the velocity data were not label dependent, Th1 or Th17 cells labeled with CFSE and CMAC were co-injected in equal proportions in preliminary experiments. No significant difference was seen in the computation of dynamic parameters or localization of Th1 and Th17 cells labeled with either CFSE or CMAC. This agrees with previous studies showing that velocity changes are not label dependent (25). Moreover, to exclude any potential interference due to the different chemical structures of the dyes, we reversed the dye combinations of Th1 and Th17 cells in every experiment.

After 48 h, the mice were anesthetized by intraperitoneal injection of ketamine (100 mg/kg body weight) and xylazine (15 mg/kg body weight) solution and prepared for surgery. A midline dorsal incision was made to expose the lumbar column over L1-L4. Laminectomy was performed with a microdrill and bone scraper to expose the spinal cord, leaving an intact dura mater. Muscles were dissected from the sides of the vertebral bone and a minor retraction of the paravertebral muscles allowed the insertion of the fine-tipped clamps of a spinal column stabilization device (Narishige STS-A Compact Spinal Cord Clamps) fitted on a customized microscope stage (Narishige MA-6N head holding adaptor on a steel base). This adaptation of an earlier method (22, 26) achieves excellent stability by combining the customized stabilization device with deep anesthesia, minimizing respiratory movements. A few drops of artificial CSF (Cold Spring Harb Protoc; 2011; doi: 10.1101/pdb.rec065730) were added without a coverglass to allow the direct immersion of the microscope objective. Blood vessels were stained by the intravenous injection of Qdot 655 fluorescent quantum dots (Molecular Probes) immediately before imaging.

### TPLM Imaging

**Figure 1:**
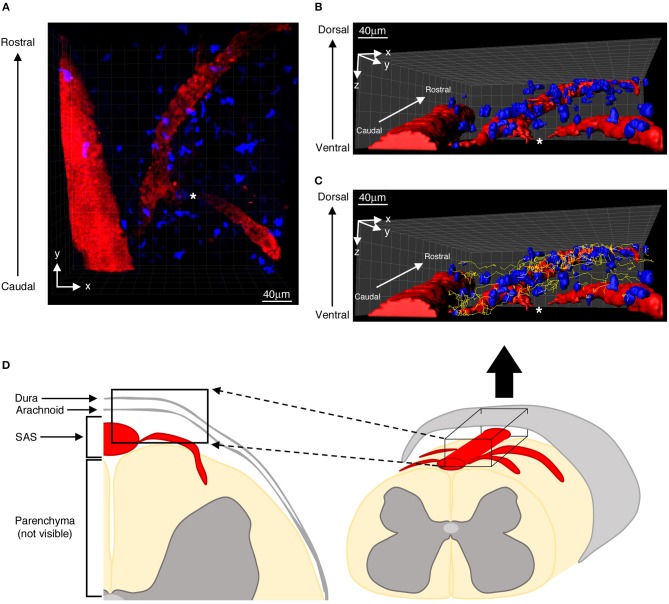
Distribution of autoreactive T helper cells in the spinal SAS of EAE mice at the disease peak. **(A)** Representative image of autoreactive Th1 cell distribution (blue) along the same focal plane of SAS pial vessels (red). Three-dimensional reconstitutions of the same image are shown without **(B)** and with **(C)** cell tracking projections (yellow lines) based on a monitoring duration of 30 min. **(D)** Schematic representation of a mouse spinal cord, highlighting the region scanned during our TPLM imaging experiments (black box). The asterisk indicates the region where the blood vessel creeps into the white matter, becoming non-detectable to our imaging system.

Mice, positioned on the stabilizing device and maintained at 37°C using a thermostatic blanket system, were placed on a customized upright Leica TCS SP5 AOBS confocal multiphoton system. The three dyes (CMAC, CFSE and Qdot 655) were excited using a mode-locked Ti:Sapphire Chameleon Ultra II laser (Coherent) and visualized with an Olympus XLUMPlanFI 20 × objective (water immersed, numerical aperture, 0.95). For imaging, we used simultaneous laser excitation at 750–800 nm. Fluorescence emission from the three dyes was separated using panchromatic electronic barrier filters and detected as red (560–650 nm), green (490–560 nm) and blue (400–500 nm) signals. Stacks of images were acquired using the Leica acquisition software. Our TPLM system setup allowed us to visualize infiltrating T cells in the whole dorsal spinal SAS thickness (Figure 1D) (12).

To create time-lapse sequences, we scanned volumes of tissue in which each plane consisted of an image of 100 × 120 μm (*x* and *y* = 2 pixels/μm), whereas *z*-stacks were acquired by taking 28 sequential steps, 2.5 μm apart for a total depth of 70 μm, at intervals of 35–40 s for up to 30 min. To increase signal contrast, we averaged two video frames for each *z*-slice. Multidimensional rendering was accomplished using Imaris software (Bitplane). In some experiments, immediately after the first acquisition of Th1 and Th17 cell behavior, 200 μl of artificial CSF containing 100 μg/ml of blocking anti-LFA-1 (clone M17/4, rat anti-mouse) or control anti-Ras (clone Y13-259, rat anti-human/mouse/ rat) monoclonal antibodies was locally applied to the exposed spinal cord and incubated for 30–60 min before a second round of imaging, to allow adequate local meningeal diffusion and dural lymphatic and CNS blood vessel recirculation (27).

### TPLM Data Analysis

TPLM data were analyzed as previously described (28). Briefly, sequences of image stacks were transformed into volume-rendered three-dimensional movies, and cell movement was analyzed using Imaris software. The three-dimensional spatial position of each cell was detected based on centroid fluorescence intensity. Cells were tracked over time manually and tracks greater than 3 min (≥12 time points) were included in the analysis. Intra-tissue tracking of sufficient numbers of cells was achieved by adhering to the following principles: (i) only fields with at least 20 fluorescent leukocytes were considered for analysis; (ii) a mean of four fields per mouse was examined; and (iii) at least 100 manually tracked cells were considered under each experimental condition. The following parameters were calculated: instantaneous and mean cell velocity, arrest index, meandering index, and cell displacement. Instantaneous velocity provides a basic parameter derived from the displacement of the cell centroid between adjacent time points. Track velocity was obtained from the mean instantaneous velocity computed from all time intervals throughout a track. The displacement of a cell moving with a constant velocity from an initial position, but randomly changing direction, is on average linearly proportional to the square root of the elapsed time. The arrest index represents the proportion of time during tracking in which a cell does not move (threshold ≤ 2 μm/min) and it was calculated from cell tracks, so the reported value represents a percentage of cells in the entire population. The meandering index was also calculated to provide another index of the directness of cell movement, based on the ratio of displacement from origin by the total path length (26).

Cytoskeletal rearrangement during cell locomotion was evaluated in terms of cell volume and cellular membrane protrusions (29, 30) using Imaris software. In particular, cellular protrusion analysis was carried out using the vertices generated in Surface Mode. This function evaluates the curvature of a surface by placing spots at each vertex and calculating curvature of these spots with respect to their neighbors (30).

### Isolation of Exogenous Infiltrating Th1 and Th17 Cells From Spinal Cord

Forty-eight hours after the transfer of CMAC-labeled Th1 or Th17 cells, C57BL/6 mice with actively-induced EAE were perfused with phosphate-buffered saline (PBS) supplemented with 1 mM Ca^2+^/Mg^2+^. The spinal cords were then carefully explanted, accurately homogenized with cold PBS using a GentleMACS Octo Dissociator (Miltenyi Biotec), washed, and incubated with 1 mg/ml collagenase (Sigma Aldrich) and 40 U/ml DNase (Thermo Fisher Scientific) for 45 min at 37°C. Cell suspensions were prepared on a 70% Percoll gradient (GE Healthcare).

### Flow Cytometry

For the assessment of cytokine production, *in vitro* differentiated Th1 and Th17 cells were stimulated for 12 h with 50 ng/ml phorbol 12-myristate 13-acetate (PMA), 1 μg/ml ionomycin, and 10 μg/ml brefeldin A. The cells were then fixed and permeabilized using the Biolegend Fixation buffer/Permeabilization wash buffer kit and labeled with phycoerythrin (PE)-conjugated rat anti-mouse IFNγ (clone REA638, Miltenyi Biotech) and eFluor660-conjugated rat anti-mouse IL-17A (clone eBio17B7, eBioscience). Control cells were stimulated under the same conditions, but without brefeldin A. For the analysis of adhesion molecule expression, *in vitro* differentiated and *ex vivo* labeled with 7-amino-4-chloromethylcoumarin (CMAC; Molecular Probes) Th1 and Th17 cells were stained with the following rat anti-mouse monoclonal antibodies: APCFire750-conjugated anti-CD49d, FITC-conjugated anti-CD11a/CD18, all from Biolegend. For *ex vivo* studies, cell viability was confirmed using 7-aminoactinomycin D (7AAD; eBioscience) and the mean fluorescence intensity (MFI) of adhesion molecule expression was calculated relative to CMAC^+^ infiltrating cells. We used a MACSQuant Analyzer (Miltenyi Biotec) for the acquisition of flow cytometry data, followed by analysis using FlowJo software.

### EAE Induction and Antibody Treatment

C57BL/6J females (8–10 weeks old) were immunized subcutaneously in the flanks with 100 μg of MOG_35−55_ peptide in 200 μl emulsion consisting of equal volumes of PBS and complete Freund's adjuvant (CFA; Difco Laboratories), supplemented with 0.8 mg/mouse *Mycobacterium tuberculosis* strain H37Ra (Difco Laboratories). Mice received 25 ng of pertussis toxin (Alexis Biochemicals) intravenously at the time of immunization and 48 h later. Clinical scores were recorded daily as previously described (28). For therapeutic anti-LFA-1 treatment, mice were injected intracisternally with 10 μl PBS containing 50 μg anti-LFA-1 antibody or the control anti-Ras monoclonal antibody. Mice were injected the day after disease onset and 4 days later. For intracisternal injection, each mouse was anesthetized with a ketamine-xylazine solution as above, the atlanto-occipital membrane was punctured with a Hamilton syringe fitted with a 27-gauge needle, and the whole antibody solution was administered in 2–3 min (31, 32). To exclude the possibility that antibody efflux from the CSF to periphery might interfere with T cell recruitment in the CNS, we also administered the anti-LFA-1 and control antibodies intravenously as a negative control.

### Immunohistochemistry Analysis

EAE mice were euthanized 3 days after the first intrathecal antibody injection and 21 days post-immunization (dpi) for neuropathology at late stages. Spinal cords were collected, frozen and used to prepare 20-μm sections for histological staining with hematoxylin/eosin and luxol fast blue to identify inflammatory infiltrates and demyelination, respectively (33). Microglia were stained using an anti-mouse ionized calcium binding adaptor molecule-1 (Iba-1) antibody (Wako) as previously described (34). All images were captured using a Leica DM6000B microscope and Leica Application Suite software. We traced the total cord area, posterior column area, gray matter area and lateral, anterolateral, and anterior column areas (mm^2^), which were manually outlined and calculated using an automated quantification system in ImageJ v1.49 (NIH) (35). Results from the quantification of neuropathological findings on 4–6 cross-sections of spinal cord per mouse taken at different lumbar and thoracic levels were expressed as a percentage of white matter area or total area.

### Immunofluorescence Analysis

For the quantification of infiltrating CD3^+^ cells, frozen 20-μm sections of spinal cord were stained with an Alexa488-conjugated anti-CD3 antibody (clone 17A2, Biolegend) diluted 1:700, with DAPI as a nuclear co-stain. All images were captured using Axio Imager Z2 (Zeiss, Germany). Acquired images were quantified using an in-house Python (https://www.python.org/) algorithm that uses the OpenCV image analysis library. Results were then represented as the area (μm^2^) of fluorescent CD3^+^ cells relative to the total spinal cord area.

### Statistics

Data are presented as means ± standard deviation (SD) or standard error of the mean (SEM). A two-tailed Student's *t*-test was used to compare two groups. A *P*-value < 0.05 was considered statistically significant. In TPLM studies, data are expressed as means ± SEM. Statistical significance was calculated using the Mann–Whitney test, with a confidence interval of 95%. In the plots of displacement and chemotactic index, we applied linear regression and compared the slopes. Outliers identification was calculated by Grubb's test with a *P*-value < 0.05 considered statistically significant.

## Results

### Th1 and Th17 Cells Differentially Infiltrate the Spinal SAS During the Phases of EAE

To study the intra-tissue behavior of effector T cells in the spinal SAS of EAE mice, we generated Th1 and Th17 cells from 2D2 TCR transgenic mice (24) and confirmed the polarization and phenotype of these cell subsets by flow cytometry (Supplementary Figure 1). TPLM analysis was carried out at different time points following immunization with the MOG_35−55_ peptide. We observed the intra-tissue behavior of labeled exogenous Th1 and Th17 cells at the following time points after immunization: (i) pre-clinical phase, 9 dpi; (ii) disease peak at 13–15 dpi; and (iii) chronic phase at 22–25 dpi. T cells were injected 48 h before visualization. In order to compare Th1 and Th17 cells within the same experiment, we used the CFSE and CMAC dye combination. Because of the potential effect of different dyes on motility, we reversed the cell type/dye combinations between observations to balance the numbers of experiments performed with each dye.

**Figure 2:**
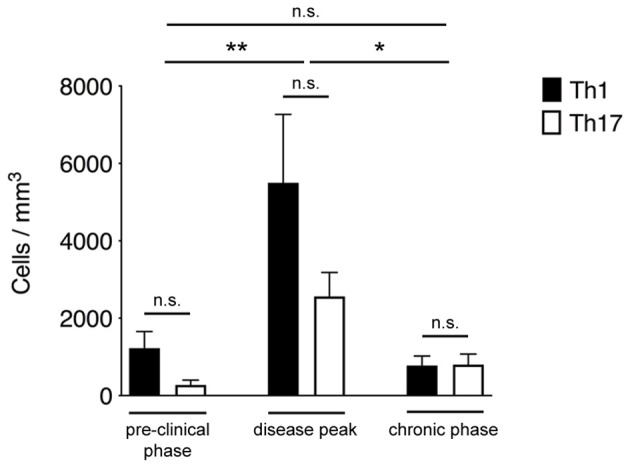
Quantification of Th1 and Th17 cell accumulation in the spinal SAS at different disease stages. MOG_35−55_-specific Th1 and Th17 cells were injected into EAE mice during the pre-clinical phase (7 dpi), at the disease peak (11–13 dpi) or during the chronic phase (20–23 dpi). TPLM imaging was performed after 48 h. Cells were quantified by manual counting in the field (volume) of acquisition. Data represent the mean ± SEM of six different fields from three independent experiments. Th1 and Th17 cells quantification for each disease stage was compared for statistical significance using the Mann-Whitney test. For comparison between cell accumulation at different disease stages Kruskal-Wallis test was applied (^*^P < 0.05; ^**^P < 0.01).

Th1 and Th17 cells showed massive infiltration (^**^P = 0.0096) of the spinal SAS at the disease peak (mean clinical score = 4.0 ± 0.5 SD) compared to the pre-clinical phase (mean clinical score = 0 ± 0 SD) (Figure 2). However, we observed a significant leakage of Qdots (the soluble vessel tracer) across the vascular wall at the same time, suggesting impairment of selective permeability in the blood–brain barrier. It was difficult to visualize cells in areas with particularly severe leakage, and such cells were therefore excluded from analysis. Although the small number of cells observed during the preclinical and chronic phases was insufficient for statistical evaluation, we observed a tendency of Th1 and Th17 cells to reduce their speed during disease progression. Taken together, these experimental observations suggest a progressive increase in T cell activation and antigen-dependent cell–cell contacts between T cells and resident cells in the spinal SAS during the clinical phases of EAE, with the chronic phase characterized by more antigen-dependent phenomena even if the number of infiltrating Th1 and Th17 cells was much lower (^*^P = 0.023) than at the disease peak (mean clinical score = 2.1 ± 0.7 SD) (Figure 2).

Particularly we found T cells spreading preferentially along the same focal plane of SAS pial vessels, with very rare cells located deeper, near to but not inside the dorsal spinal cord white matter, probably crawling on leptomeningeal pial membrane as previously described (12) (Figure 1). Moreover, the myelinated nature of the CNS parenchyma limits TPLM penetration, hampering the visualization of potential T cells inside the spinal cord white matter (17, 36). Given this technical limitation, TPLM studies on T cell motility in white and gray matter parenchyma would require *ex vivo* acute spinal cord explants (15).

### Th1 and Th17 Cells Show Differential Dynamics in the Spinal SAS of EAE Mice at the Disease Peak

The preliminary experiments described above indicated that exogenous pro-inflammatory Th1 and Th17 cells massively infiltrate the spinal SAS at the disease peak, with significant differences between cell types in terms of motility behavior. To better understand these differences, we investigated Th1 and Th17 behavior in more detail in the inflamed spinal SAS during the disease peak. Three-dimensional reconstitution allowed us to discriminate between endothelium-extravasated cells and those crawling on the luminal surface of the vessels. We found very few cells crawling on the luminal surface of the vessels, and we excluded these cells from further analysis.

**Figure 3:**
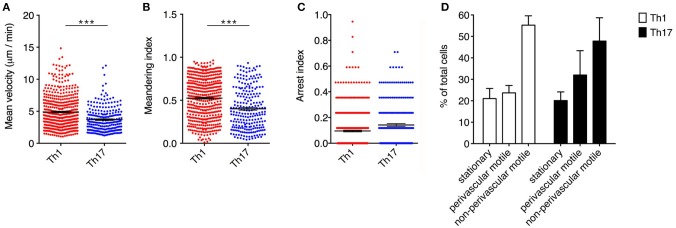
Dynamics of Th1 and Th17 cell movement inside the spinal SAS at the EAE disease peak. Exogenous myelin-specific autoreactive Th1 and Th17 cells were injected into EAE mice at the disease peak (11–13 dpi). TPLM imaging was performed on the exposed spinal cord 48 h later. The mean velocity **(A)**, meandering index **(B)** and arrest index **(C)** were calculated to compare the behavior of infiltrating cells. Data represent the mean ± SEM from a minimum of nine to a maximum of 490 cells from three independent experiments (^***^P < 0.0001). **(D)** Comparison of the dynamic behavior of the Th1 and Th17 populations. Data represent the mean ± SEM of six different fields from three independent experiments.

Notably, Th1 cells showed a significantly higher velocity than Th17 cells (mean = 4.9 μm/min for Th1 cells but 3.7 μm/min for Th17 cells, *P* < 0.0001) (Figure 3A). Also, the significantly higher meandering index of Th1 cells (mean = 0.52 for Th1 cells and 0.40 for Th17 cells, *P* < 0.0001) suggested an increase in directional bias (Figure 3B). Although the arrest index was significantly higher in Th17 cells (0.1 for Th1 cells and 0.14 for Th17 cells, *P* < 0.0001) (Figure 3C). We defined three subsets of extravasated cells: (i) stationary cells, comprising all cells moving at a mean velocity ≤ 2 μm/min; (ii) perivascular motile cells, comprising the cells moving close to the vascular environment following the vessel wall, although among the cells moving along the abluminal vascular surface we were unable to distinguish cells moving between the endothelium and basement membrane (perivascular cuff) from cells that were completely extravasated; and (iii) non-perivascular motile cells, specifically non-perivascular cells that were dispersed in the SAS environment. Our analysis showed that ~80% of the Th1 and Th17 cells displayed rapid movement, whereas 20% were stationary. These latter cells were distributed preferentially close to the vessel wall, anchored around a fixed point, suggesting physical contacts with other resident cells, or moving slowly in a looping pattern within a local area, behavior that was previously termed “swarming” (18) (Figure 3D). Among the motile Th1 cells, we distinguished a cell population showing perivascular movement (20%), whereas the rest of the cells were dispersed, moving inside the SAS (60%). The Th17 cells showed similar behavior, with ~30% of cells moving close to the vessel walls and 50% moving in the SAS environment (Figure 3D).

**Figure 4:**
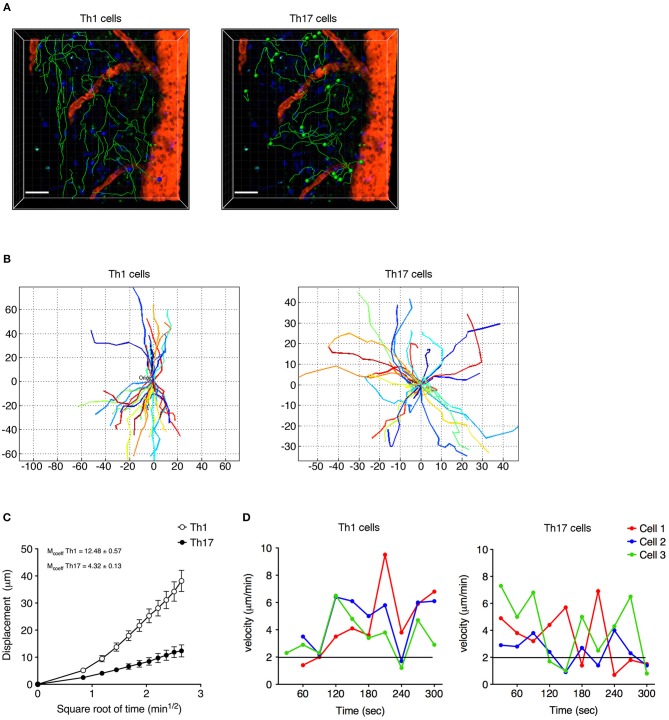
Non-perivascular motile Th1 and Th17 cell dynamics in the spinal SAS of EAE mice at the disease peak. **(A)** Representative images of autoreactive Th1 and Th17 cells moving in a dispersed manner in the SAS of EAE mice at the disease peak. Cell tracks (green lines) are also shown (scale bar = 50 μm; red/orange color = blood vessel). Time projections of 30 min are shown. **(B)** Normalized trajectories of non-perivascular motile Th1 and Th17 cells. **(C)** Mean displacement of Th1 and Th17 cells. Data represent the mean ± SEM of at least 30 cells from three independent experiments. **(D)** Instantaneous velocities of three representative Th1 cells (left panel) and Th17 cells (right panel). Cells moving at <2 μm/min were considered non-motile.

Analyzing the cells dispersed in the SAS in more detail, we observed that Th1 cells moved in a straight line with a characteristic rostro-caudal axis displacement, covering long distances in both directions, whereas the Th17 cells exhibited more restrained migration trajectories with a preference for radial movement (Figures 4A,B and Supplementary Movies 1, 2). By plotting the displacement of these cells against the square root of time, we found a linear plot for Th1 cells compatible with directed migration, whereas Th17 cells displayed a plateau suggesting constrained motility (Figure 4C). Furthermore, the instantaneous velocity data revealed that Th1 cells meandered directly through the tissue with steady movement, whereas Th17 cells showed stop-and-go behavior as they migrated in the tissue (Figure 4D). Collectively, these data suggest that the Th1 cells covered a greater area than the Th17 cells.

### Th1 and Th17 Cells Show Differential Expression of LFA-1 in the Spinal SAS of EAE Mice at the Disease Peak

**Figure 5:**
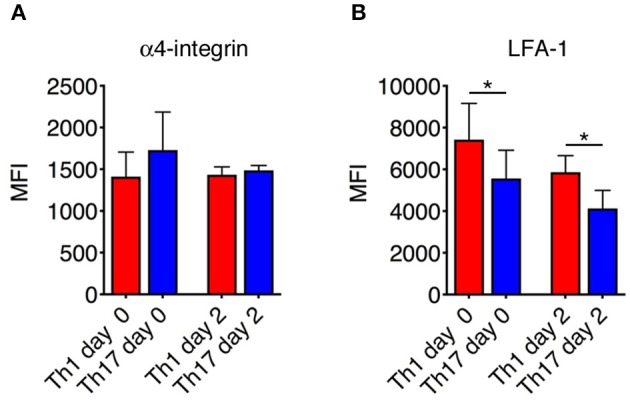
Integrin expression on Th1 and Th17 cells. The expression of α4 integrins **(A)** and LFA-1 **(B)** on Th1 and Th17 was evaluated both *in vitro* before injection and *ex vivo* on exogenous CMAC^+^ T cells isolated from spinal cords 48 h after adoptive transfer. Samples were analyzed by flow cytometry. The mean fluorescence intensity (MIF) of LFA-1 was statistically higher on Th1 cells than Th17 cells both before injection and 48 h after cell transfer. Data represent the mean ± SD of four mice per condition from two independent experiments (^*^P < 0.05).

The differential motility behavior of Th1 and Th17 cells during the EAE disease peak indicated that different molecular mechanisms are used by these cells to migrate in the spinal SAS. To determine whether the contrasting dynamic behaviors of these T cell subsets reflect distinct adhesive patterns, we compared the expression of α4 integrins and LFA-1 in exogenous *in vitro* differentiated Th1 and Th17 cells by flow cytometry immediately before injection and inside isolated spinal cords of EAE-induced mice 2 days after adoptive transfer at the disease peak. We observed no differences in α4 integrin expression between Th1 and Th17 cells (Figure 5A), but LFA-1 was more abundant on Th1 cells both when comparing cultured populations (MIF = 7423 in Th1 vs. 5560 in Th17cells, ^*^P = 0.0159) and when comparing adoptively transferred cells isolated from the spinal cords of recipient mice (MIF = 5866 in Th1 vs. 4127 in Th17cells, ^*^P = 0.0286) (Figure 5B). Interestingly, LFA-1 expression in the *in vitro* differentiated Th1 and Th17 cells was not affected *in vivo* by the spinal cord microenvironment (Figure 5B). The differential expression of LFA-1 in Th1 and Th17 cells potentially reflects their distinct kinetics, related to the immune cell phenotype.

### Blocking LFA-1 Affects the Motility Behavior and Deformability of Moving Th1 Cells Dispersed in the Spinal SAS

**Figure 6:**
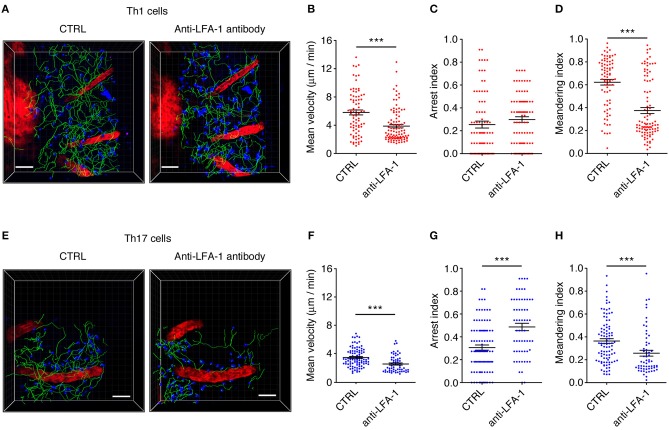
An LFA-1 blockade differently affects autoreactive Th1 and Th17 cell dynamics in the spinal SAS at the EAE disease peak. Th1 and Th17 cell dynamics were analyzed in the spinal SAS of EAE mice at the disease peak before (CTRL) and after the application of anti-LFA-1 antibodies to the exposed spinal cord. **(A)** Representative images of Th1 cells moving in the spinal SAS before and after antibody treatment. **(B)** The mean velocity, **(C)** arrest index and **(D)** meandering index of the Th1 cell population in the spinal SAS in the presence or absence of the anti-LFA-1 antibody (^***^P < 0.0001). **(E)** Representative images of Th17 cells moving in the spinal SAS before (CTRL) and after antibody treatment. **(F)** The mean velocity, **(G)** arrest index and **(H)** meandering index of Th17 cells in the presence or absence of the anti-LFA-1 antibody (^***^P < 0.0001). **(A,E)** Cell tracks (green lines) are also shown (scale bar = 50 μm; red color = blood vessel). Time projections of 30 min are shown. In all graphs, data represent the mean ± SEM of 100 cells from three independent experiments.

**Figure 7:**
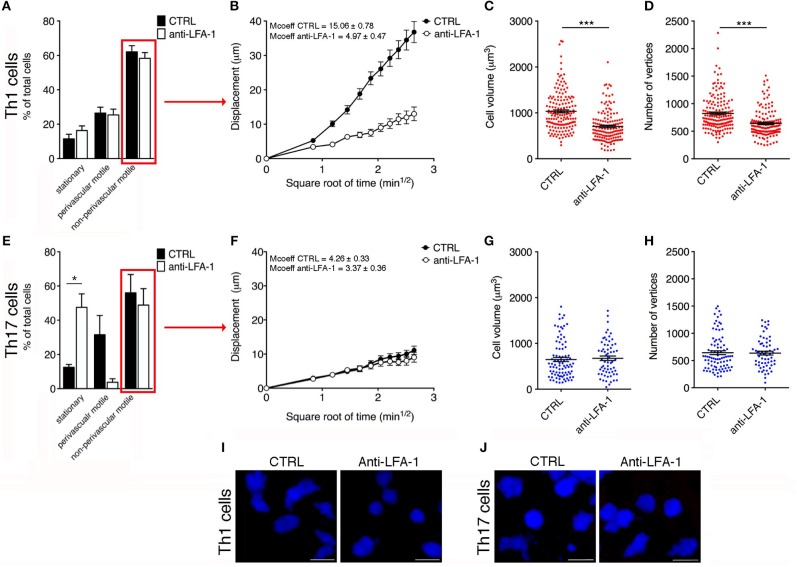
Blocking LFA-1 specifically affects non-perivascular motile Th1 cell dynamics and cell deformability in the SAS at the EAE disease peak. **(A)** Dynamic properties of Th1 subpopulations are not affected by blocking LFA-1. **(B)** Mean displacement of non-perivascular motile Th1 cells is reduced by a LFA-1 blockade. The volume **(C)** and number of vertices **(D)** of non-perivascular motile Th1 cells differ before (CTRL) and after the inhibition of LFA-1 (^***^P < 0.0001). **(E)** The dynamic properties of the Th17 population: the number of perivascular motile Th17 cells is reduced following the application of an anti-LFA-1 antibody (^*^P < 0.05). **(F)** Mean displacement of non-perivascular motile Th17 cells in the tissue is not affected after antibody treatment. The volume **(G)** and number of vertices **(H)** of non-perivascular motile Th17 cells do not change after LFA-1 inhibition. **(I)** Representative images of non-perivascular motile Th1 cells before and after anti-LFA-1 treatment. Note the presence of highly-polarized cells before antibody application (left panel) and the predominant rounded cell morphology induced by blocking LFA-1 (right panel) (scale bar = 15 μm). **(J)** Blocking LFA-1 had no effect on non-perivascular motile Th17 cell cytoskeletal organization, compared to controls (scale bar = 15 μm). **(B,F)** Data represent the mean ± SEM of 100 cells from three independent experiments. **(C,D,G,H)** Data represent the mean ± SEM of 200 cells from three independent experiments.

We next investigated the potential role of integrin LFA-1 in the regulation of Th1 cell motility in EAE mice at the disease peak (mean clinical score = 4.3 ± 1.1 SD) by the direct application of a blocking anti-LFA-1 antibody (clone M17/4) to the exposed spinal cord. The inhibition of LFA-1 activity reduced the mean velocity of Th1 cells from 5.7 to 3.8 μm/min (*P* < 0.0001) (Figures 6A,B, and Supplementary Movies 3, 4), whereas treatment with the control antibody had no effect (Supplementary Figure 2). Interestingly, we observed no significant change in the arrest index (from 0.25 to 0.30, *P* = 0.0959) after treatment, suggesting that Th1 cells were still moving but at a lower velocity (Figure 6C). The meandering index was substantially lower after blocking LFA-1 (falling from 0.62 to 0.37, *P* < 0.0001) (Figure 6D), suggesting that LFA-1 controls the capacity of Th1 cells to move in a straight line. Accordingly, we did not observe a significant shift in the percentage of stationary cells (from 11.4 to 16.3%, *P* > 0.05) (Figure 7A). Furthermore, displacement analysis focusing on the portion of moving Th1 cells dispersed in the SAS (non-perivascular motile cells) showed a significant reduction in the curve slope after blocking LFA-1 (*P* < 0.0001), suggesting that Th1 cells were no longer able to move over long distances covering much of the tissue volume (Figure 7B). Overall, these data suggest that LFA-1 is needed for Th1 cells to move in a straight line inside the spinal SAS at the EAE disease peak.

Cell motility is a complex cellular process that involves reorganization of the cytoskeleton. This reorganization includes a dynamic rearrangement of the cortical actin cytoskeleton, which is required for the development of cellular protrusions (including lamellipodia and filopodia) and stress fibers. Cytoskeletal rearrangement, in particular actin dynamics, is induced via integrin by Rho-family GTPases (37). One possible explanation for the dramatic impact of blocking LFA-1 activity on Th1 cell motility in the spinal SAS is the potential role of this integrin in the cell deformation and cytoskeletal rearrangements necessary for T cell migration. To test this hypothesis, we compared the morphology of non-perivascular motile Th1 cells before and after anti-LFA-1 antibody treatment. Generally, the initial response of a cell to a migration-promoting agent is to polarize and extend protrusions in the direction of migration. These protrusions can be large, broad lamellipodia or spike-like filopodia, which can be quantified by counting the number of vertices in each cell (30). Following LFA-1 inhibition, the volume of Th1 cells was significantly lower compared to the cells in untreated mice (704 vs. 1032 μm^3^, *P* < 0.0001) and similarly the number of vertices decreased (639 vs. 823, *P* < 0.0001) (Figures 7C,D,I), indicating that treated Th1 cells moving in a dispersed manner in the spinal SAS become more rounded and lower in volume, presumably because blocking LFA-1 interferes with the cytoskeleton. Taken together, these data suggest that blocking LFA-1 has a strong impact on Th1 cell dynamics in the spinal SAS during EAE, probably by disrupting cell deformability and the outside-in signaling controlling cytoskeletal dynamics.

### LFA-1 Controls the Motility Behavior of Perivascular Th17 Cells

We next investigated the role of LFA-1 in the SAS dynamics of Th17 cells during the EAE disease peak (mean clinical score = 4.2 ± 1.4 SD). As observed for Th1 cells, blocking LFA-1 affected the dynamics of Th17 cells, reducing their mean velocity from 3.5 to 2.6 μm/min (*P* < 0.0001) (Figures 6E,F and Supplementary Movies 5, 6). However, unlike Th1 cells, a large proportion of Th17 cells became completely immotile following the LFA-1 blockade. Indeed, we observed a strong increase in the arrest index from 0.31 to 0.49 (*P* < 0.0001) (Figure 6G) with a corresponding increment in the portion of non-motile cells from 12.5 to 47.5% (*P* < 0.05) (Figure 7E). The meandering index of Th17 cells decreased after the inhibition of LFA-1 from 0.36 to 0.26 (*P* < 0.0001) (Figure 6H). Because the meandering index of Th17 cells was lower than that of Th1 cells (Figure 3B), the reduced meandering index of Th17 cells after blocking LFA-1 was probably due to an increase in the proportion of non-motile cells rather than changes in the motility behavior of moving cells.

The analysis of moving Th17 cells dispersed in the spinal SAS after antibody treatment suggested that blocking LFA-1 did not affect the motility behavior of these cells, because the mean displacement did not change (Figure 7F). Moreover, compared to Th1 cells, the non-perivascular motile Th17 cells appeared more rounded and less polarized due to their slower movement (30) and, as expected, the volume and number of vertices of these cells was unchanged after treatment (Figures 7G,H,J). These data clearly show that LFA-1 controls the movement of perivascular Th17 cells in the spinal SAS during EAE, but does not affect either the velocity or deformability of non-perivascular motile Th17 cells.

### Intrathecal Injection of a Blocking Anti-LFA-1 Antibody Inhibits the Development of EAE

**Figure 8:**
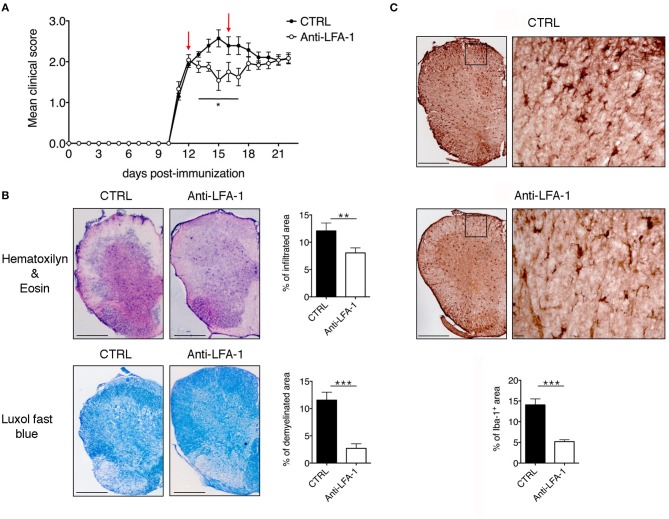
Intrathecal injection of an anti-LFA-1 blocking antibody inhibits EAE progression in MOG_35−55_-immunized mice. **(A)** C57BL/6-immunized mice were injected with 10 μl PBS containing 50 μg of control antibody (CTRL) (rat anti-human Ras, clone Y13259) or anti-LFA-1 blocking antibody. The mice were injected in the cisterna magna the day after disease onset (11–13 dpi) and 4 days later (red arrows) and were then followed until 22 dpi and scored daily for the severity of clinical disease symptoms. Data represent the mean ± SEM of 16 mice per condition from four independent experiments (^*^P < 0.05). **(B)** Neuropathology of EAE mice treated with the anti-LFA-1 blocking antibody. Mice were euthanized 3 days after the first antibody injection (14 dpi), and spinal cords were analyzed for the presence of inflammatory infiltrates and demyelination by hematoxylin/eosin and luxol fast blue staining, respectively. Representative consecutive spinal cord sections from mice treated with control (CTRL) or anti-LFA-1 antibodies are shown (4–6 cross sections of spinal cord per mouse, *n* = 3 mice). Quantitative analysis of the infiltrated cells and demyelination area for each condition. Error bars indicate SEM (^**^P < 0.005; ^***^P < 0.0005). Scale bar = 200 μm. **(C)** Representative images of Iba-1^+^ microglia in mice treated with a control or anti-LFA-1 antibodies. Quantitative analysis of microglial cells for each condition to determine the area occupied by Iba-1^+^ cells (4–6 cross sections of spinal cord per mouse*, n* = 3 mice per condition). Error bars indicate SEM (^***^P < 0.0005). Scale bar = 200 μm (left) or 20 μm (right).

**Figure 9:**
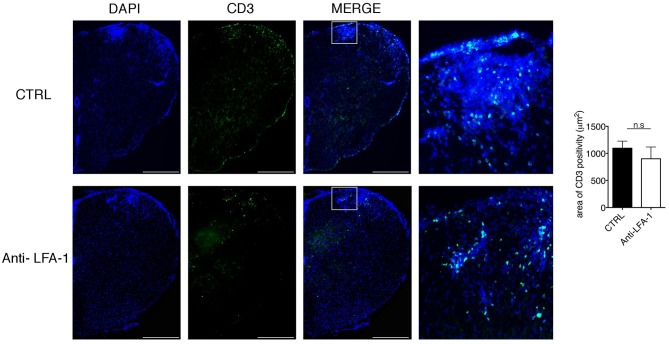
Intrathecal injection of an anti-LFA-1 blocking antibody inhibits EAE progression T cells without eliminating CD3+ T cells in MOG_35−55_-immunized mice. Mice were euthanized 3 days after the first antibody injection (14 dpi), and spinal cords were analyzed for the presence of CD3+ infiltrated cells by immunofluorescence staining. Representative spinal cord sections from mice treated with control (CTRL) or anti-LFA-1 antibodies are shown (4–6 cross sections of spinal cord per mouse, *n* = 3 mice). Quantitative analysis of the area occupied by CD3^+^ cells. Error bars indicate SEM. Scale bar = 200 μm (left). Magnification of the merged quadrant is shown in the right-hand images.

The motility of effector T cells in the SAS is regulated by resident leptomeningeal cells and is necessary for their reactivation and their pro-inflammatory role (11–14). For this reason, we hypothesized that interfering with the local dynamics of T cells in the spinal SAS may limit their pathogenicity and may ameliorate EAE symptoms in immunized mice. To test this hypothesis, we treated MOG_35−55_-immunized EAE mice on the day after disease onset and 4 days later by intrathecal injection with an anti-LFA-1 antibody or a control antibody. We found that the local blocking of LFA-1 achieved the rapid and significant inhibition of disease compared to the control group (Figure 8A, ^*^P < 0.05). Disease amelioration was associated with a significant decline in both inflammatory cell infiltration (^**^P = 0.0034) and demyelination (^***^P = 0.0003) in the anti-LFA-1 treated animals (Figure 8B). Furthermore, the massive microgliosis in the lumbar spinal cord of mice injected with the control antibody was attenuated in the animals treated with anti-LFA-1, suggesting that the intrathecal injection of the blocking antibody achieved a profound reduction in the severity of CNS inflammation (Figure 8C, ^***^P < 0.0001). A second anti-LFA-1 injection further delayed the appearance of the clinical peak in EAE mice (Figure 8A). Moreover, the non-significant reduction in the quantity of infiltrating CD3^+^ T cells in the lumbar spinal cords of mice treated with the anti-LFA-1 antibody (*P* = 0.073) suggested that the inhibition of LFA-1 blocks the function of T cells without eliminating them (Figure 9). However, 5–6 days after the cessation of treatment, the symptoms of EAE emerged and the mice reached a clinical score comparable to control animals (Figure 8A). The mice treated with the anti-LFA-1 antibody tended to display minor neuropathological features at the late stage of EAE (21 dpi) (Supplementary Figure 3). Moreover, except for inflammatory infiltrating cells (*P* = 0.029) (Supplementary Figure 3A), we observed no significant differences between the two groups of treated animals. Notably, the comparison of early and late EAE revealed that the neuropathology (in terms of infiltrates and demyelination) following anti-LFA-1 antibody treatment was preserved until disease chronicity, whereas in mice treated with the control antibody, the amelioration of clinical symptoms was associated with a tendency of reduction in inflammatory cell infiltration (12.08 ±1.44 at 14 dpi vs. 8.18 ± 1.17 at 21 dpi, *P* = 0.143) and demyelination (11.55 ± 1.45 at 14 dpi vs. 2.58 ± 0.66, ^***^P = 0.0002) (Supplementary Table 1).

Our intrathecal injection technique could potentially trigger the rapid efflux of antibody from the CSF into the peripheral lymphatic system and circulation, partly due to the increased pressure in the CSF and to the massive leakage at the peak of the disease. To rule the possibility that antibody efflux interfered with leukocyte recruitment during the progression of EAE, we also treated the MOG_35−55_-immunized EAE mice with the anti-LFA-1 or control antibodies by intravenous injection, with the same administration dose and timing used for intrathecal treatment. We observed no significant differences in EAE progression between the anti-LFA-1 and control mice (Supplementary Figure 4). Collectively, these results suggest that interfering with the local dynamics of effector T cells can help to reduce their pathogenic potential and may therefore offer a useful therapeutic strategy to control the progression of autoimmune diseases.

## Discussion

Cells of the immune system communicate constantly with the extracellular environment by releasing and sensing soluble factors or through direct physical interactions with surrounding cells. These processes are significant because they influence cell proliferation, survival and effector functions. In particular, the ability of T cells to scan APCs in secondary lymphoid organs and to show dynamic behavior in the tissue parenchyma of target organs is essential for the generation and regulation of inflammatory responses (38). Recent studies using TPLM have shown that the leptomeninges represent a key checkpoint for T-cell infiltration into the CNS during autoimmune inflammation (11–14, 17). Following extravasation, T cells move on the abluminal vascular surface and pial membrane, scanning the area for phagocytes that present antigens. These contacts stimulate the effector T cells to produce pro-inflammatory mediators, trigger tissue invasion and guide infiltrating autoimmune T cells into the CNS parenchyma (12, 17).

We used TPLM to visualize the spinal SAS and investigated the dynamic motility of autoreactive Th1 and Th17 cells during EAE, the murine model of MS. Our data revealed a previously unappreciated difference in the behavior of these two effector T cell populations suggesting that their pathogenic roles may depend on their ability to move in the spinal SAS and to contact resident cells.

Although there is evidence for the involvement of Th1 and Th17 cells during the pathogenesis of MS and EAE (5, 9, 39–41), the molecular mechanisms involved as these cells contribute to the induction and progression of demyelinating diseases are not well-understood. Importantly, the balance or predominance of Th1 and Th17 responses in MS patients is correlated with the heterogeneity of disease symptoms, variations in clinical course, and the localization of CNS lesions (42–44). Importantly, the physiological and pathological functions associated with particular phenotypes of activated T cells, such as the production of certain transcription factors and cytokines, are known to be reversible (45).

It is now clear that studies in mouse models and humans, showing the importance of Th1 or Th17 cells based solely on the detection of their cytokines, are complicated by the plasticity of activated T cell subsets. For example, committed cells producing IL-17 readily convert to cells that produce IFNγ during the course of EAE (46). In our experimental setting, the phenotype of transplanted Th1 and Th17 cells differentiated *in vitro* remained stable in the CNS. Importantly, we found that differences in LFA-1 integrin expression between Th1 and Th17 cells before adoptive transfer cells were maintained after 48 h of *in vivo* migration, strongly suggesting that the phenotype of our cells does not change *in vivo* during the first 2 days after cell transfer.

The movement of Th1 and Th17 cells inside the spinal cord during the pre-clinical phase of actively-induced EAE is unclear. Our results revealed only a few exogenous T cells infiltrating the spinal SAS during the pre-clinical phase, in agreement with previous reports showing limited injury to the blood–spinal cord barrier during this phase (47, 48). Our results are also supported by the data from a transfer EAE model in Lewis rats, which revealed that most of the autoreactive transfected T cells were distributed between the blood and peripheral lymphoid organs during the prodromal phase (17, 49). We found that effector T cells were not uniformly distributed in the tissue volume we analyzed and observed small groups of both cell subsets in specific areas of the spinal SAS, suggesting preferential entry points. The rare activated cells that initially invade the CNS are able to create a permissive environment, which is probably not dependent on antigen presentation, setting the stage for the second inflammatory wave, which contains antigen-specific cells as well as other immune system cells with no antigen specificity (50).

At the disease peak, we observed the massive infiltration of Th1 and Th17 cells, with 20% of both populations in a stationary state in close proximity to the vessel wall, suggesting physical contacts with perivascular APCs (11–13). TPLSM imaging allowed the visualization of dynamic contacts between T cells and perivascular phagocytes, revealing that activation signaling is not sufficient to completely arrest autoreactive T cells in the SAS, and suggesting that contacts with APCs in the leptomeningeal area are necessary to guide the infiltrating autoreactive T cells into the CNS parenchyma, rather than causing their prolonged arrest (13). Among the portion of motile Th1 and Th17 cells, it was possible to distinguish between the majority of cells moving distantly from the vessels, dispersed in the SAS structures of the spinal cord, and a smaller number of cells that were following the vessel wall (perivascular motile cells). The dynamic nature of perivascular immune cells has previously been reported in the CNS during EAE, indicating that CD4^+^ T cell compartmentalization along CNS vessels is dependent on the chemokine CXCR4, suggesting that the process is actively promoted and may facilitate the efficient and rapid screening of perivascular professional APCs (51). We found that Th17 cells displayed more constrained stop-and-go motility, whereas Th1 cells moved directly along the axis of the spinal cord parallel to the main central vein, suggesting a rostro-caudal biased walk with confined lateral movement (52). This previously unreported difference between Th1 and Th17 cell motility behavior suggests that these populations play distinct roles during the induction of EAE (42, 53).

Compared to mesenchymal and epithelial cells, leukocytes (including T cells) exhibit a peculiar migration behavior known as amoeboid migration. Three biomechanical factors facilitate amoeboid cell motility: integrin-mediated adhesion, leading edge protrusion driven by actin polymerization, and myosin II-mediated contractility (54). Due to their amoeboid movement, T cells can navigate through many tissues and organs, including blood vessels, secondary lymphoid organs, and peripheral tissues, to perform their immune surveillance and immune response functions (55). CD4^+^ T cell motility is actively promoted in the CNS during EAE. Pre-existing scaffolds guide lymphocyte migration in lymphoid tissues, and specialized structures are induced in the leptomeningeal area during CNS inflammation to guide T cell migration, potentially facilitating the screening of APCs and integrating relevant stimulatory costimulatory, and regulatory signals. The arachnoid membrane, trabeculae and pia mater, which make up the SAS, are always immersed in CSF and must be exposed to its immunological influence. Chronic inflammation may trigger the maturation of immunostimulatory trabecular fibroblast-like cells in organized ectopic aggregates, commonly described as tertiary lymphoid tissues (56). Moreover, during CNS inflammation, these trabecular fibroblast-like cells could regulate immune cell entry into the SAS from the bloodstream and control the transition of inflammatory responses from regions proximal to CNS barriers into the parenchyma (57). Notably, chronic inflammatory conditions during MS and EAE induce the organization of a lymphoid-like tissue in which fibroblastic networks secrete extracellular matrix (ECM) components, chemokines and cytokines and upregulate ICAM-1 expression (58–62). Recent studies suggest a role for integrins as receptors for stromal cells and the ECM in neurological disorders, because they represent an important link between the ECM, the intracellular cytoskeleton, and signaling pathways (63).

Our data show that integrin LFA-1 is necessary, in different ways, for Th1 and Th17 cell trafficking in the spinal SAS during the EAE disease peak. Interestingly, anti-LFA-1 treatment significantly reduced the rate of Th1 cell movement and affected their directed movement in the SAS, while increasing the number of perivascular non-motile Th17 cells. The strong impact of LFA-1 inhibition on T cell dynamics in the SAS is supported by earlier reports showing that both LFA-1 and its ligand ICAM-1 (expressed on dendritic cells) are required for the rapid interstitial migration of lymphocytes in the lymph node parenchyma (21). Similar results were recorded following the pretreatment of T cells with inhibitors of actin organization, indicating that cytoskeletal rearrangement is required for the rapid, directed motility of T cells in lymph nodes (21). Our cellular morphology data revealed that Th1 cells lost volume and the capacity to extend cellular protrusions during their movement when LFA-1 was inhibited, suggesting that LFA-1 is actively involved in the cytoskeletal rearrangements necessary for amoeboid migration (64). On the other hand, integrins were found to be unnecessary for amoeboid movement maintained by the presence of chemokines, which support the movement despite the absence of adhesive interactions (65). However, in our study, non-perivascular motile Th1 cells moved in a straight line, but in both directions, which does not indicate the presence of a chemotactic factor.

The LFA-1 blockade did not change the motility or morphology of the moving Th17 cells dispersed in the SAS environment, even though these cells were moving at a slower velocity than the Th1 cells. In such a scenario, we propose that the selective inhibitory effect on the motility of Th1 cells may reflect the greater abundance of LFA-1 on Th1 compared to Th17 cells. Moreover, the LFA-1 blockade dramatically inhibited the movement of Th17 cells in close proximity to the perivascular environment, so the role of LFA-1 in the motility of these cells remains unclear and further experiments are required to understand which adhesion molecules are involved. We speculate that blocking LFA-1 could interfere with the sub-endothelial crawling of Th17 cells along pericytes as demonstrated for neutrophils. Indeed, the abluminal crawling of neutrophils is supported by ICAM-1 (expressed by pericytes) and its leukocyte integrin ligands, Mac-1 and LFA-1 (66). Moreover, stimulated trabecular fibroblast-like cells were shown to enhance the expression of ICAM-1, perhaps facilitating their interaction with incoming extravasated Th17 cells (59, 67). ICAM-1 is expressed on the trabecular network, the cellular surface lining the CSF system, and seems to promote leukocyte migration independently of the vascular system (67).

A recent study described the inhibition of intraluminal crawling T cells following the intravenous co-application of anti-LFA-1 and anti-VLA-4 antibodies 2 days after adoptive transfer, when the majority of T cells are still closely related to the vessels wall, with only 2% of the cells detached from vessels (12). However, in our study, we demonstrate for the first time that LFA-1 plays a key role in the motility of Th1 cells inside SAS after extravasation from the pial vessels, probably as these cells are attempting to reach the CNS parenchyma.

Our experiments revealed that the intrathecal injection of an anti-LFA-1 antibody after the onset of EAE has a significant impact on disease progression, suggesting that interfering with the motility of activated T cells in the CNS could offer a new approach for the treatment of autoimmune diseases such as MS. However, LFA-1 is also required for the generation of a functional immunological synapse during antigen presentation to T lymphocytes by APCs in secondary lymphoid organs (68). This process may also be important during T cell reactivation by local APCs in the CNS (15), and the confined inhibition of LFA-1 in the CNS of EAE mice may also limit effector T cell reactivation in the CNS parenchyma. Furthermore, TPLM analysis has shown that Th17 cells form synapse-like contacts with neurons in a process dependent on LFA-1/ICAM-1, leading to neuronal dysfunction and axonal damage during EAE (69). LFA-1 is also implicated in the sequestration and accumulation of activated T cells in the leptomeningeal milieu, a key process required for the infiltration of T cells into the CNS parenchyma during EAE (70). Therefore, we cannot exclude the possibility that local anti-LFA-1 treatment might affect not only T cell motility in the spinal SAS, but also T cell reactivation and contacts with leptomeningeal and neural cells, as well as other immunological and non-immunological processes inside the CNS. Moreover, the disease returns with equal severity once the treatment period is over, suggesting that the local LFA-1 blockade exerts a protective mechanism (71, 72). Further studies are therefore required to clarify the effects of anti-LFA-1 treatment on CNS local inflammatory responses during EAE.

Integrins are valuable therapeutic targets for the treatment of several inflammatory and autoimmune diseases, but the severe side effects associated with systemic treatment have led to the discontinuation of several clinical trials (73, 74). In MS, the anti-α_4_ integrin antibody natalizumab is currently indicated for the treatment of relapsing-remitting forms of the disease, even though in some patients it causes the reactivation of John Cunningham (JC) virus in the CNS, probably due to interference with immune surveillance mechanisms (75). The intrathecal administration of certain therapeutic proteins is therefore emerging as an alternative to systemic therapy. For example, intrathecal administration has been used to deliver enzymes as a treatment for brain storage diseases in rodents and humans, Nogo-A protein in stroke models, and trastuzumab in patients with breast cancer brain metastases (76). The intrathecal injection of antibodies interfering with effector T cell dynamics in the CNS may complement existing MS therapies and, if administered at the onset of relapse, may lead to a faster resolution and therefore inhibit chronic disease progression, without impairing systemic immunity.

Taken together, our results suggest that LFA-1 is needed to control effector T cell motility in the spinal cord under the inflammatory conditions of the EAE disease peak. As well as improving the understanding of immune cell involvement in neurological diseases, our data suggest that local anti-LFA-1 antibodies could also be used for the treatment of neurological and autoimmune diseases.

## Data Availability Statement

The datasets generated for this study are available on request to the corresponding author.

## Ethics Statement

All animal experiments were approved by the Italian Ministry of Health, Department of Veterinary Public Health, Nutrition and Food Safety, Directorate General of Animal Health and Veterinary Medicine, as required by Italian legislation (D. Lgs 26/2014, application of European Directive 2010/63/EU). Protocol numbers 33588 and 30969 were assigned for the approved animal experiments. All animal experiments were supervised by the local Institutional Animal Care Committee (OPBA) of the University of Verona and were conducted according to current European Community rules.

## Author Contributions

SD, BR, SA, EZ, VD, AA, EP, NL, TC, and GA performed the research. GT, FP, and BR analyzed the data. BR and GC designed the research and wrote the paper.

### Conflict of Interest

The authors declare that the research was conducted in the absence of any commercial or financial relationships that could be construed as a potential conflict of interest.

## Supplementary Material

Supplementary Figure 1 Cytokine production by Th1 and Th17 cells. IFNγ and IL-17A production by Th1 and Th17 cells was evaluated on the day of cell injection in EAE recipient mice. Samples were analyzed by flow cytometry. Approximately 40% of Th1 cells produced IFNγ and more than 40% of Th17 cells produced IL-17A with negligible INFγ production.

Supplementary Figure 2 Effect of a control antibody on Th1 and Th17 motility in the spinal SAS of EAE mice at the disease peak. Th1 and Th17 cell motility behavior was evaluated in the spinal cord at the disease peak before and after the administration of a control antibody (rat anti- human Ras, clone Y13259). Antibody administration did not affect the velocity **(A,D)**, motility **(B,E)**, and meandering index **(C,F)** of either the Th1 or Th17 cells. Data in all graphs represent the mean ± SEM of 50–100 cells from two independent experiments.

Supplementary Figure 3 Neuropathology of late stage EAE in MOG_35−55_-immunized mice following the intrathecal injection of an anti-LFA-1 blocking antibody. **(A)** Immunized C57BL/6 mice were injected with 10 μl PBS containing 50 μg of a control antibody (CTRL) (rat anti-human Ras, clone Y13259) or an anti-LFA-1 blocking antibody. The mice were injected in the cisterna magna the day after disease onset (11-13 dpi) and 4 days later. **(A)** Quantification of neuropathology of EAE mice treated with the anti-LFA-1 blocking antibody. Mice were euthanized 21 dpi and spinal cords were analyzed for the presence of inflammatory infiltrates **(A)**, CD3^+^ T cells **(B)**, demyelination **(C)**, and Iba-1^+^ microglia **(D)**. Error bars indicate SEM (^*^*P* < 0.05).

Supplementary Figure 4 Intravenous injection of an anti-LFA-1 blocking antibody does not significantly affect EAE progression in MOG_35−55_-immunized mice. Immunized C57BL/6 mice were injected intravenously with 200 μl PBS containing 50 μg of a control antibody (CTRL) (rat anti- human Ras, clone Y13259) or an anti-LFA-1 blocking antibody. The mice were injected the day after disease onset (11-13 dpi) and 4 days later (red arrows) and were then followed until 22 dpi and scored daily for the severity of clinical disease symptoms. Data represent the mean ± SEM of eight mice per condition. The intravenous anti-LFA-1 antibody administered at the same dose used for the intrathecal treatment did not significantly affect EAE progression during the observation period.

Supplementary Movie 1 Non-perivascular motile Th1 cell dynamics in the SAS. Representative tracks of MOG_35−55_-specific Th1 cells (blue cells) moving in the meningeal spinal cord structures of MOG_35−55_-immunized mice at the EAE disease peak (clinical score = 4). This video shows how Th1 cells move in straight lines covering long distances in the spinal cord meningeal structures. Vascular permeability is visualized by the leakage of red dye into the extravascular space, as indicated by the yellow ring. Vessels are shown in red. Scale bar = 50 μm.

Supplementary Movie 2 Non-perivascular motile Th17 cell dynamics in the SAS. Representative tracks of MOG_35−55_-specific Th17 cells (green cells) moving in the meningeal spinal cord structures of MOG_35−55_-immunized mice at the EAE disease peak (clinical score = 4). This video shows how Th17 cells display more constrained migration. Vessels are shown in red. Vascular permeability is visualized by the leakage of red dye into the extravascular space, as indicated by the yellow ring. Scale bar = 50 μm.

Supplementary Movies 3 and 4 Th1 cells moving in the SAS before and after anti-LFA-1 treatment. These videos show representative tracks of total MOG_35−55_-specific Th1 cells (blue cells) moving inside spinal cord leptomeninges of MOG_35−55_-immunized mice at the EAE disease peak (clinical score = 4) before (movie 3) and after (movie 4) the local administration of an anti-LFA-1 antibody. Blocking LFA-1 led to a reduction in Th1 cell velocity, interfering with their straight-line motility. Notably, non-perivascular motile Th1 cells were mainly affected, whereas the motility of perivascular Th1 cells was unaffected. Vessels are shown in red. Scale bar = 50 μm.

Supplementary Movies 5 and 6 Th17 cells moving in the SAS before and after anti-LFA-1 treatment. These videos show representative tracks of total MOG_35−55_-specific Th17 cells (blue cells) moving inside the spinal cord leptomeninges of MOG_35−55_-immunized mice at the EAE disease peak (clinical score = 4) before (movie 5) and after (movie 6) the local administration of an anti-LFA-1 antibody. Blocking LFA-1 mainly affected the dynamics of perivascular motile Th17 cells, resulting in a substantial loss of movement. Vessels are shown in red. In movie 6, vascular permeability is visualized by the leakage of red dye into the extravascular space, as indicated by the yellow ring. Scale bar = 50 μm.

Supplementary Table 1 Neuropathology of EAE mice treated intrathecally with the anti-LFA-1 blocking antibody. Mice were euthanized 3 days after the first antibody injection (14 dpi) and during chronic phase (21 dpi). Spinal cords were analyzed for the presence of inflammatory infiltrates, demyelination, and Iba-1^+^ microglia. Results are expressed in mean ± SEM of 4-6 cross sections of spinal cord per mouse. (*n* = 3 mice per condition). Statistics were calculated using the Mann–Whitney test, with a confidence interval of 95%.
