# Supplementary figures and images for: LFA-1 Controls Th1 and Th17 Motility Behavior in the Inflamed Central Nervous System
Source: Front Immunol. 2019 Oct 18;10:2436. doi: 10.3389/fimmu.2019.02436 (PMC6813462; doi:10.3389/fimmu.2019.02436)

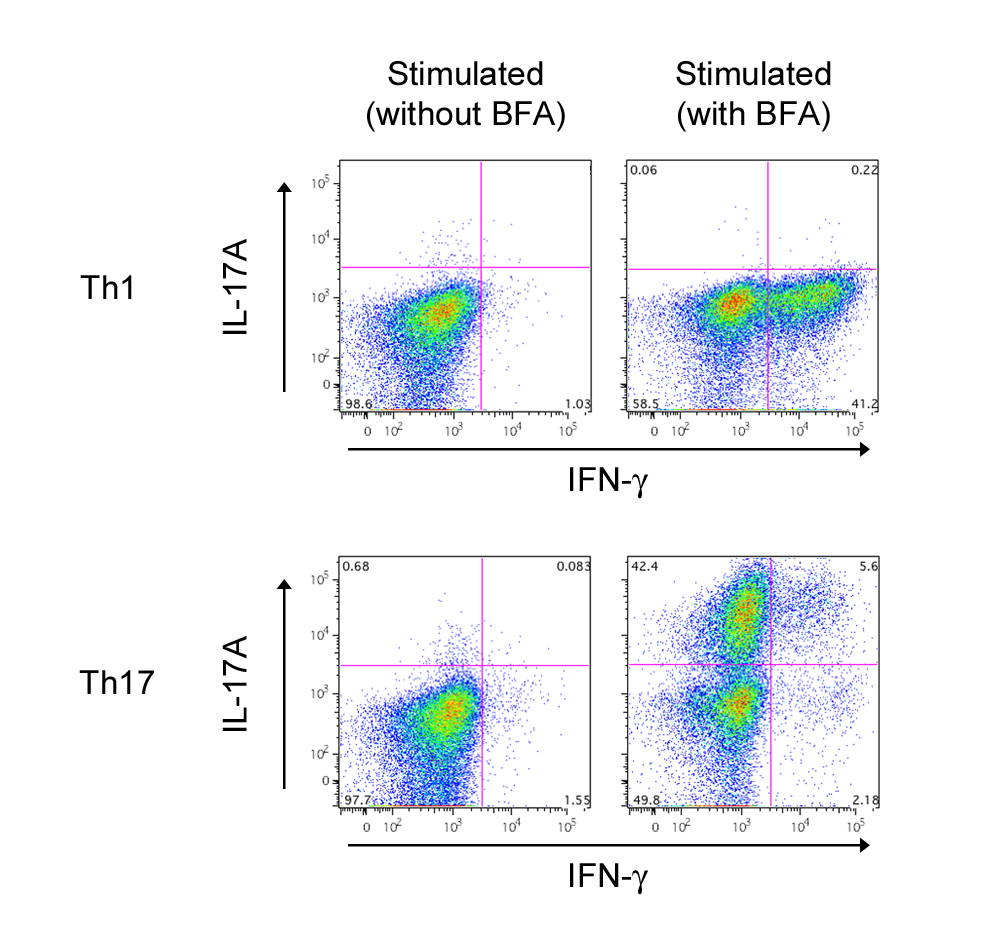

Supplement: Supplementary Figure 1 — Cytokine production by Th1 and Th17 cells. IFNγ and IL-17A production by Th1 and Th17 cells was evaluated on the day of cell injection in EAE recipient mice. Samples were analyzed by flow cytometry. Approximately 40% of Th1 cells produced IFNγ and more than 40% of Th17 cells produced IL-17A with negligible INFγ production. [file Image_1.JPEG]

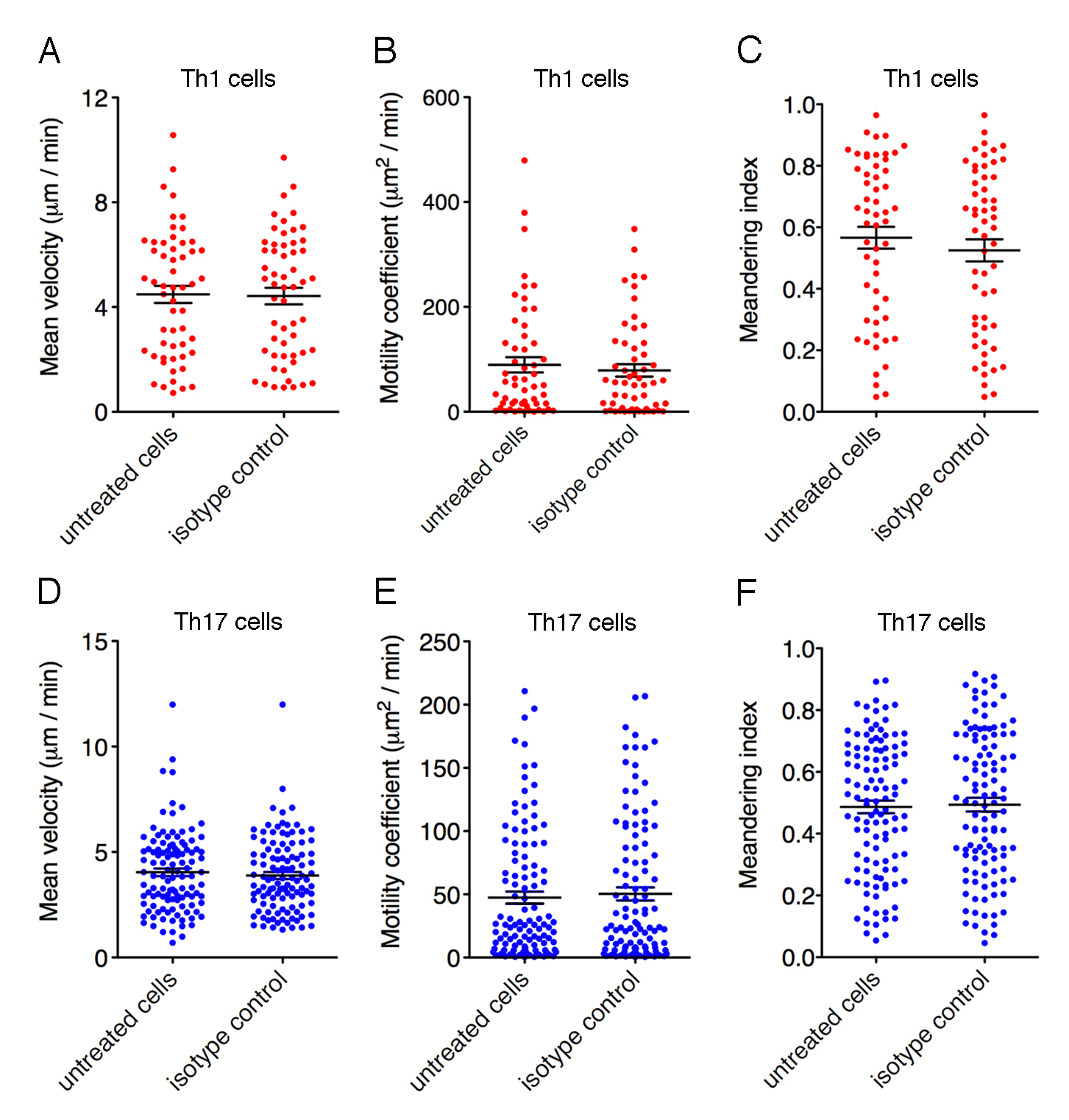

Supplement: Supplementary Figure 2 — Effect of a control antibody on Th1 and Th17 motility in the spinal SAS of EAE mice at the disease peak. Th1 and Th17 cell motility behavior was evaluated in the spinal cord at the disease peak before and after the administration of a control antibody (rat anti- human Ras, clone Y13259). Antibody administration did not affect the velocity (A,D), motility (B,E), and meandering index (C,F) of either the Th1 or Th17 cells. Data in all graphs represent the mean ± SEM of 50–100 cells from two independent experiments. [file Image_2.JPEG]

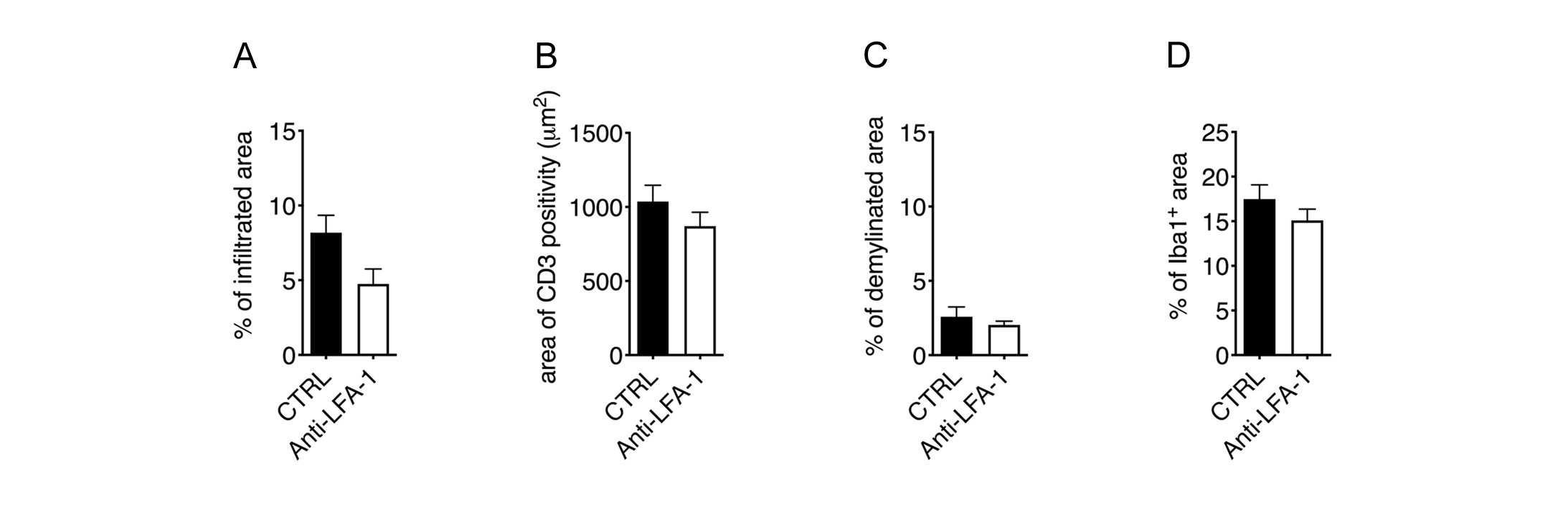

Supplement: Supplementary Figure 3 — Neuropathology of late stage EAE in MOG35−55-immunized mice following the intrathecal injection of an anti-LFA-1 blocking antibody. (A) Immunized C57BL/6 mice were injected with 10 μl PBS containing 50 μg of a control antibody (CTRL) (rat anti-human Ras, clone Y13259) or an anti-LFA-1 blocking antibody. The mice were injected in the cisterna magna the day after disease onset (11-13 dpi) and 4 days later. (A) Quantification of neuropathology of EAE mice treated with the anti-LFA-1 blocking antibody. Mice were euthanized 21 dpi and spinal cords were analyzed for the presence of inflammatory infiltrates (A), CD3+ T cells (B), demyelination (C), and Iba-1+ microglia (D). Error bars indicate SEM (*P < 0.05). [file Image_3.JPEG]

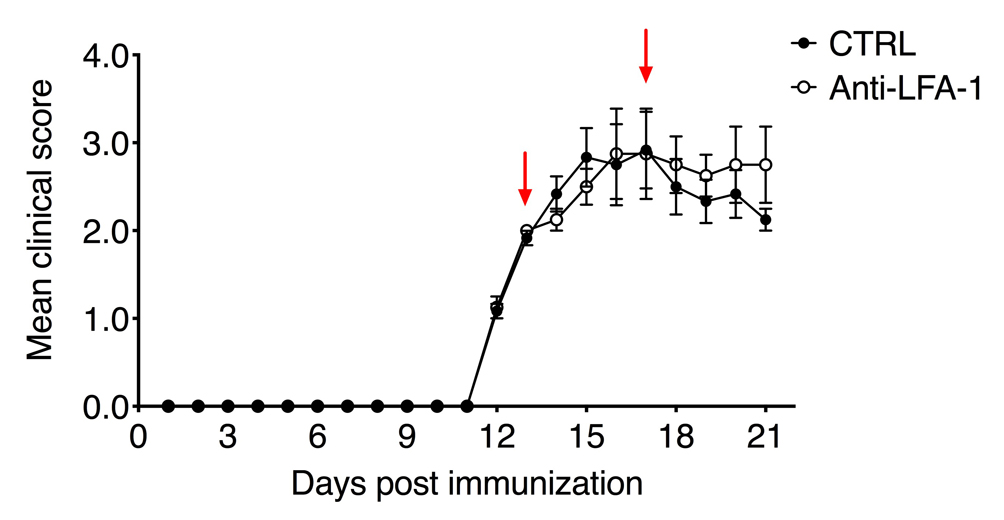

Supplement: Supplementary Figure 4 — Intravenous injection of an anti-LFA-1 blocking antibody does not significantly affect EAE progression in MOG35−55-immunized mice. Immunized C57BL/6 mice were injected intravenously with 200 μl PBS containing 50 μg of a control antibody (CTRL) (rat anti- human Ras, clone Y13259) or an anti-LFA-1 blocking antibody. The mice were injected the day after disease onset (11-13 dpi) and 4 days later (red arrows) and were then followed until 22 dpi and scored daily for the severity of clinical disease symptoms. Data represent the mean ± SEM of eight mice per condition. The intravenous anti-LFA-1 antibody administered at the same dose used for the intrathecal treatment did not significantly affect EAE progression during the observation period. [file Image_4.JPEG]
