# Supplementary material for: LFA-1 Controls Th1 and Th17 Motility Behavior in the Inflamed Central Nervous System
Source: Front Immunol. 2019 Oct 18;10:2436. doi: 10.3389/fimmu.2019.02436 (PMC6813462; doi:10.3389/fimmu.2019.02436)
Supplement: Supplementary Table 1 — Neuropathology of EAE mice treated intrathecally with the anti-LFA-1 blocking antibody. Mice were euthanized 3 days after the first antibody injection (14 dpi) and during chronic phase (21 dpi). Spinal cords were analyzed for the presence of inflammatory infiltrates, demyelination, and Iba-1+ microglia. Results are expressed in mean ± SEM of 4-6 cross sections of spinal cord per mouse. (n = 3 mice per condition). Statistics were calculated using the Mann–Whitney test, with a confidence interval of 95%. [file Table_1.pdf]

Table I. Neuropathology of EAE mice treated intrathecally with the anti-LFA-1 blocking antibody. Mice were euthanized 3 days after the first antibody injection (14 dpi) and during chronic phase (21 dpi). Spinal cords were analyzed for the presence of inflammatory infiltrates, demyelination and Iba-1$^+$ microglia. Results are expressed in mean ± SEM of 4–6 cross sections of spinal cord per mouse. (*n* = 3 mice per condition). Statistics were calculated using the Mann–Whitney test, with a confidence interval of 95%.

**Neuropathological feature**		**14 dpi**	**21 dpi**	***P* value**
**Infiltrates (%)**	**CTRL**	12.08 ± 1.44	8.18 ± 1.17	P=0.143
	**Anti-LFA-1**	8.05 ± 0.92	4.75 ± 1	P=0.052
**Demyelination (%)**	**CTRL**	11.55 ± 1.45	2.58 ± 0.66	***P=0.0002
	**Anti-LFA-1**	2.71 ± 0.83	2.03 ± 0.26	P=0.938
**Iba-1 (%)**	**CTRL**	14.07 ± 1.43	17.48 ± 1.60	P=0.084
	**Anti-LFA-1**	5.18 ± 0.48	15.09 ± 1.27	****P<0.0001
